# Sonic Hedgehog Regulates Osteoblast Function by Focal Adhesion Kinase Signaling in the Process of Fracture Healing

**DOI:** 10.1371/journal.pone.0076785

**Published:** 2013-10-04

**Authors:** Yuu Horikiri, Tsuyoshi Shimo, Naito Kurio, Tatsuo Okui, Kenichi Matsumoto, Masahiro Iwamoto, Akira Sasaki

**Affiliations:** 1 Department of Oral and Maxillofacial Surgery, Okayama University Graduate School of Medicine, Dentistry, and Pharmaceutical Sciences, Okayama, Japan; 2 Division of Orthopedic Surgery, the Children’s Hospital of Philadelphia Research Institute, Philadelphia, Pennsylvania, United States of America; University of Alabama at Birmingham, United States of America

## Abstract

Several biological studies have indicated that hedgehog signaling plays an important role in osteoblast proliferation and differentiation, and sonic hedgehog (SHH) expression is positively correlated with phosphorylated focal adhesion kinase (FAK) Tyr^397^. However, the relationship between them and their role in the process of normal fracture repair has not been clarified yet. Immunohistochemical analysis revealed that SHH and pFAK Tyr^397^ were expressed in bone marrow cells and that pFAK Tyr^397^ was also detected in ALP-positive osteoblasts near the TRAP-positive osteoclasts in the fracture site in the ribs of mice on day 5 after fracture. SHH and pFAK Tyr^397^ were detectable in osteoblasts near the hypertrophic chondrocytes on day 14. *In vitro* analysis showed that SHH up-regulated the expression of FAK mRNA and pFAK Tyr^397^ time dependently in osteoblastic MC3T3-E1 cells. Functional analysis revealed that 5 lentivirus encoding short hairpin FAK RNAs (shFAK)-infected MC3T3-E1 cell groups displayed a round morphology and decreased proliferation, adhesion, migration, and differentiation. SHH stimulated the proliferation and differentiation of MC3T3-E1 cells, but had no effect on the shFAK-infected cells. SHH also stimulated osteoclast formation in a co-culture system containing MC3T3-E1 and murine CD11b^+^ bone marrow cells, but did not affect the shFAK-infected MC3T3-E1 co-culture group. These data suggest that SHH signaling was activated in osteoblasts at the dynamic remodeling site of a bone fracture and regulated their proliferation and differentiation, as well as osteoclast formation, via FAK signaling.

## Introduction

Fracture healing is a complex physiological process that involves the combination of both intramembranous and endochondral ossification. The osteoblasts and osoteoclasts play a crucial role in this process. For bone formation to occur, osteoblast cells must proliferate and migrate from the bone marrow compartment to bone surfaces, where they adhere, differentiate, and deposit the bone matrix concurrently with bone and bony callus resorption by osteoclasts [1].

Sonic hedgehog (Shh) is a 45-kDa potent signaling protein that regulates the proliferation, differentiation, and cellular patterning across a wide range of cell types [2,3]. It has been shown that hedgehog signaling is involved in fracture healing and bone maintenance [4,5]. In the initial stages of fracture repair, the expression of sonic hedgehog is detected in proliferating callus-forming cells in the periosteum [6]. It was reported that hedgehog proteins directly act on osteogenic precursor cells and osteoblasts to stimulate osteogenic differentiation [7]. Additionally, the implantation of Shh-transduced cells increased the bone regeneration in a rabbit model of calvaria defects [8]. On the other hand, Mark et al showed that conditional deletion of Ptch selectively in mature osteoblasts enhances hedgehog signaling and leads to increased osteoclastogenesis [9]. They also showed that hedgehog signaling indirectly induce osteoclast formation by upregulating parathyroid hormone-related peptide (PTHrP), which promoted receptor activator for nuclear factor κB ligand (RANKL). SHH stimulates osteoclast formation with PTHrP in a co-culture system consisting of ST2 cells and murine CD11b+ bone marrow cells [10]. These reports suggest that Shh has a osteogenic and osteoclastogenic activity in osteoblast cells [11], but the downstream signaling of SHH in fracture healing has not been determined.

Focal adhesion kinase (FAK) is a 125-kD non-receptor tyrosine kinase that plays a major role in mediating signal transduction by integrins, as well as by growth factor receptors, in the regulation of cell adhesion, migration, proliferation, and differentiation in a variety of cell types [12,13,14]. The role of FAK in bone formation and remodeling is unclear, because FAK-deficient embryonic mice die at E8.5-E9.0 [15]. A recent report showed that the phosphorylation of FAK is critical for bone formation and osteoblast migration [16]. FAK deficiency in osteoblasts and osteocytes *in vivo* results in delayed bone healing and remodeling and interrupts the response of bone marrow cells to anabolic mechanical stimuli in a tibial injury model [17,18]. Phosphorylated FAK at the Tyr^397^ site is a critical factor for the adhesion and migration of osteoblast in fracture healing [19]. A novel FAK Tyr^397^ inhibitor suppresses osteoblast proliferation and differentiation, as well as osteoclast formation, through PTHrP-induced RANKL expression in murine bone stromal ST2 cells [20]. However, little is known about the regulation of FAK during bone healing.

In this study, we examined the distribution patterns of SHH and FAK phosphorylated at its Tyr^397^ during fracture healing, and determined the functional effect of SHH-associated FAK on the osteoblasts in this process.

## Materials and Methods

### Cell lines and culture conditions

Murine preosteoblast cell line MC3T3-E1 was obtained from the RIKEN BioResource Center Cell Bank (Tsukuba, Japan). Primary cultures of mouse CD11b^+^ bone marrow cells were incubated in Modified Eagle Medium (αMEM). Both cell types were cultured in an atmosphere of 10% CO_2_ at 37°C.

### Infection with short-hairpin RNA

To generate MC3T3-E1 cells expressing short-hairpin (sh) RNA specific for FAK, we transfected the cells with lentiviral particle vectors separately bearing 5 kinds of shRNA: shFAK#1, CCGGCGAGTATTAAAGGTCTTTCATCTCGAGAATGAAAGACCTTTAATACTCGTTTTT; shFAK#2, CCGGCCAACCTTAATAGAGAAGAAACTCGAGTTTCTTCTCTATTTAAGGTTGGTTTTT; shFAK#3, CCGGGCCTTAACAATGCGTCAGTTTCTCGAGAAACTGACGCATTGTTAAGGCTTTTT; shFAK#4, CCGGCCTGGCATCTTTGATATTATACTCGAGTTTCTTCTCTATTTAAGGTTGGTTTTT; and shFAK#5, CCGGCGGTCCAATGACAAGGTATATCTCGAGATATACCTTGTCATTGGACCGTTTTT. The cells were infected according to the manufacturer’s protocol (Sigma), FAK and pFAK Tyr^397^ knockdown was confirmed by performing immunoblot analysis and evaluated by using ImageJ.

### Purification of osteoclast progenitors

Bone marrow cells were washed twice by centrifugation in 20 ml of cold buffer containing sterile phosphate-buffered saline (PBS) supplemented with 0.5% bovine serum albumin (Sigma, St. Louis, MO, USA) and 2 mM EDTA (Sigma). The cell pellet was resuspended in 80 µl of buffer per 10^7^ cells, and the cells were magnetically labeled by adding 20 µl of anti-CD11b microbeads per 10^7^ cells. The cells were next incubated for 30 min on ice and then washed by centrifugation with a volume of buffer 10-fold that of the labeling volume and resuspended in 500 µl of buffer per 10^8^ cells. CD11b^+^ cells were isolated by using an MD depletion column (Miltenyi Biotec Inc, Bergisch Gladbach, Germany) placed in the magnetic field of a MidiMACS separation unit (Miltenyi Biotec Inc.).

### Cell proliferation assay

MC3T3-E1 cells were plated in a 96-well plate at 5 × 10^3^ cells per well. The MTS assay was performed to obtain a relative cell number after 24 hours of incubation under the experimental procedure specified by the manufacturer (Cell Titer 96 AQueous One Solution Cell proliferation Assay; Promega, Madison, WI, USA).

### RNA extraction and reverse-transcription PCR

Total RNA was isolated by using an RNeasy Minikit (QIAGEN, Tokyo, Japan). Complementary DNA was generated from 1 µg of total RNA in a final volume of 20 µl by use of a first-strand cDNA synthesis kit (Takara, Tokyo, Japan), and then amplified for 30 cycles with the following oligonucleotide primer pairs: 5’-AAGCTCATCGGCAGTGTGGAC -3’ and 5’-GGTATGGGACGCTGTGAGCA -3’ for FAK, 5’-TGCTTCCTCGCTGCTGGTGT -3’ and 5’-CCGTGTTTTCCTCATCCTTA -3’ for Shh, 5’-ACTCATCCCTATGGCTCGTG -3’ and 5’-GGTAGGGAGCTGGGTTAAGG -3’ for osterix, 5’-AATGGGCGTCTCCACAGTAAC -3’ and 5’-CTGAGTGGTGTTGCATCGC -3’ for ALP, 5’-TTCTCCAACCCACGAATGCAC -3’ and 5’- CAGGTACGTGTGGTAGTGAGT -3’ for Runx2, 5’-TCTGATGAGACCGTCACTGC -3’ and 5’-AGGTCCTCATCTGTGGCATC-3’ for osteopontin (OPN), 5’-GGTCGGGCAATTCTGAATT -3’ and 5’-ACATCTAGGACATCCATGC -3’ for RANKL, 5’-ACCAAAGTGAATGCCGAGAG -3’ and 5’-TCTGTGGTGAGGTTCGAGTG-3’ for osteoprotegerin (OPG), and 5’-TGAACGGGAAGCTCACTGG-3’ and 5’-TCCACCACCCTGTTGCTGTA -3’ for glyceraldehyde-3-phosphatase dehydrogenase (GAPDH). Each PCR cycle was carried out for 30 s at 94 °C, 30s at 55 °C, and 1 min at 68 °C. The PCR products were then separated on 2% agarose gels containing ethidium bromide and visualized under ultra violet light. The expected sizes of PCR products were 1894 bp for FAK, 242 bp for SHH, 238 bp for OSX, 1289 bp for ALP, 108 bp for Runx2, 852 bp for OPN, 204 bp for RANKL, 215 bp for OPG, and 307 bp for GAPDH.

### Immunoblot analysis

MC3T3-E1 cells were rinsed once with ice-cold PBS and lysed in an ice-cold lysis buffer (50 mM Tris-HCl, pH 7.4, containing 150 mM NaCl, 1% Triton X-100, 1% NP-40, 10 mM NaF, 100 mM leupeptin, 2 mg/ml aprotinin, and 1 mM phenylmethyl sulfonyl fluoride). The cell lysates containing 10 µg of total protein in lysis buffer were electrophoresed in 12% sodium dodecyl sulfate polyacrylamide gel electrophoresis (SDS-PAGE) gels, and the proteins were transferred to nylon membranes (Immobilon-P; Millipore Co.), which were then incubated with primary and secondary antibodies according to the ECL chemiluminescence protocol (RPN2109, Amersham Biosciences, Buckinghamshire, UK) to detect secondary antibody binding. Anti-FAK and FAK p-Tyr^397^ antibodies were purchased from BD Biosciences (San Jose, CA, USA). Actin antibodies were purchased from Santa Cruz Biotechnology (Santa Cruz, CA, USA) and used at a 1:200 dilution. Horseradish peroxidase-conjugated anti-mouse and anti-rabbit immunoglobulins (IgG) were used as the secondary antibodies at a 1:1000 dilution.

### Immunofluorescence analysis

MC3T3-E1 cells grown on 8-well chamber slides (BD Falcon, Bedford, MD, USA) were fixed with 4% paraformaldehyde in PBS for 20 min and then permeabilized with 0.2% Triton X-100 in PBS for 5 min at room temperature. The cells were then incubated in a blocking solution (3% bovine serum albumin in PBS) for 30 min and thereafter for 90 min at room temperature with affinity-purified mouse anti-human phosphorylated-FAK Tyr^397^ as primary antibody, diluted in 1% bovine serum albumin-PBS. After having been washed 3 times with PBS, the cells were incubated for 1 h with rabbit anti-mouse IgG antibody labeled with FITC as the secondary antibody. For actin staining, fixed cells were incubated with rhodamine-conjugated phalloidin (Invitrogen Corporation, Grand Island, NY, USA) for 1 h at room temperature. After having been washed twice with PBS, the cells were counterstained for 5 min at room temperature with 4′6′-diamidino-2-phenylindole diluted to 1:1000 with PBS. The slides were finally mounted with Vectashield (Vector Laboratories Ltd, Peterborough, UK) and viewed under a fluorescence microscope (IX81, Olympus, Tokyo, Japan).

### Adhesion assay

MC3T3-E1 cells cultured in medium including 10% serum were trypsinized and diluted to a concentration of 1 X 10^5^ cells/ml with the same medium. Then 100 µl of cells was added to each well of a 96-well plate, and the cells were incubated for 1 h. The wells were then flooded with additional DMEM and placed bottom up for 15 min at room temperature. After discarding of the floating cells, the attached cells were fixed with 4% paraformaldehyde and stained with methylene blue; and the number of attached cells was examined by microscopy.

### Scratch assay

MC3T3-E1 cells were grown to confluence in 6-well tissue culture dishes, and a single scratch was then made in the confluent monolayer by using the tip of a sterile 200-µl pipette. The monolayer was washed with PBS, after which complete medium containing SHH (500 nM) or vehicle alone was added. Serial photographs of the same scratched section were taken after 48 hours. The number of cells that had migrated over the margins of the wounds was counted at 12, 24, and 48 h after scratch treatment.

### Alkaline phosphatase and Alizarin red staining

For estimation of ALPase activity, MC3T3-E1 cells was grown to confluence in 24-well multiplates with αMEM containing 10% FBS, 50 µg/ml ascorbic acid, and 10 mM β-glycerophosphate (β-GP). Then, the cells were either kept in the same medium or the medium was exchanged for fresh αMEM + 10%FBS and incubated for 5 days. After the incubation, the cells were rinsed with PBS; and then the enzyme reaction was initiated by the addition of 0.5 mg/ml naphthol AS-BI (Sigma) and Fast Red trisodium salts (Sigma) in 50 mM Tris-HCl (pH9.5). The reaction proceeded at 37°C for 2 min. For estimation of mineralization, MC3T3-E1 cells were cultured in complete αMEM containing 10% FBS, 50 µg/ml ascorbic acid, and 10 mM β-GP for 10 days. The cells were subsequently fixed in 95% ethanol for 15 min at 37°C, washed with PBS, and stained with 1% alizarin red for 5 min at room temperature. Thereafter, they were viewed under an inverted microscope; and the stained area was measured by using imaging software (Lumina Vision /OL).

### TRAP staining

For osteoclast differentiation, 1 x 10^5^ murine CD11b^+^ bone marrow cells/well were plated onto a monolayer of 1 x 10^4^ MC3T3-E1 cells/well cells in a 96-well plate with or without 10 nM PTHrP or the desired amount of SHH. After 8 days of incubation, the cells were then fixed and stained for tartrate-resistant acid phosphatase (TRAP) activity (Sigma); and then the number of TRAP-positive multinucleate cells in each well was counted.

### Experimental model for fracture healing

The right eighth rib of male ICR mice (5 week old, 22 ± 2 g body weight) was fractured [21]. Briefly, each mouse was anesthetized, and the eighth rib on the right side was exposed and cut vertical to the axis with scissors. For a sham operation as a control, the right eighth rib of another mouse was similarly exposed but not fractured. The experimental protocols were approved by the Ethics Review Committee for Animal Experimentation of the Okayama University Graduate School of Medicine, Dentistry and Pharmaceutical Sciences.

### Histochemical and immunohistochemical analysis of surgically resected samples

The sections were sequentially dewaxed by passage through a series of xylene, graded ethanol, and water immersion steps. After having been autoclaved in 0.2% citrate buffer for 15 min, the sections were incubated with 3% hydrogen peroxide for 30 min to block endogenous peroxidase activity. Next, they were incubated with a 1:200 dilution of antibodies against FAK p-Tyr^397^ (mouse IgG, BD biosciences) or SHH (mouse IgG, R&D systems) overnight at 4°C followed by 3 washes with Tris-buffered saline (TBS). The slides were then treated with a streptoavidin-biotin complex; Envision System Labeled Polymer, horseradish peroxidase (HRP; Dako, Carpinteria, CA, USA) for 60 minutes at a dilution of 1:100. The immunoreaction was visualized by using 3,3'-diaminobenzidine (DAB) substrate-chromogen solution (Dako Cytomation Liquid DAB Substrate Chromogen System; Dako), and counterstaining was performed with hematoxylin. For the detection of TRAP and ALP activities, a TRAP/ALP staining kit was used according to the manufacturer’s protocol (Wako, Osaka, Japan). Finally, the sections were immersed in an ethanol and xylene bath and then mounted for examination.

### Statistical analysis

Data were analyzed by using the unpaired Student’s t-test for the analysis of 2 groups or one-way ANOVA for the analysis of repeated multiple group comparisons. Results were expressed as the mean ± S.D. *P < 0.05 and ** P < 0.01 were considered statistically significant.

## Results

### Expression of SHH and pFAK Tyr^397^ in fractured ribs

**Figure 1:**
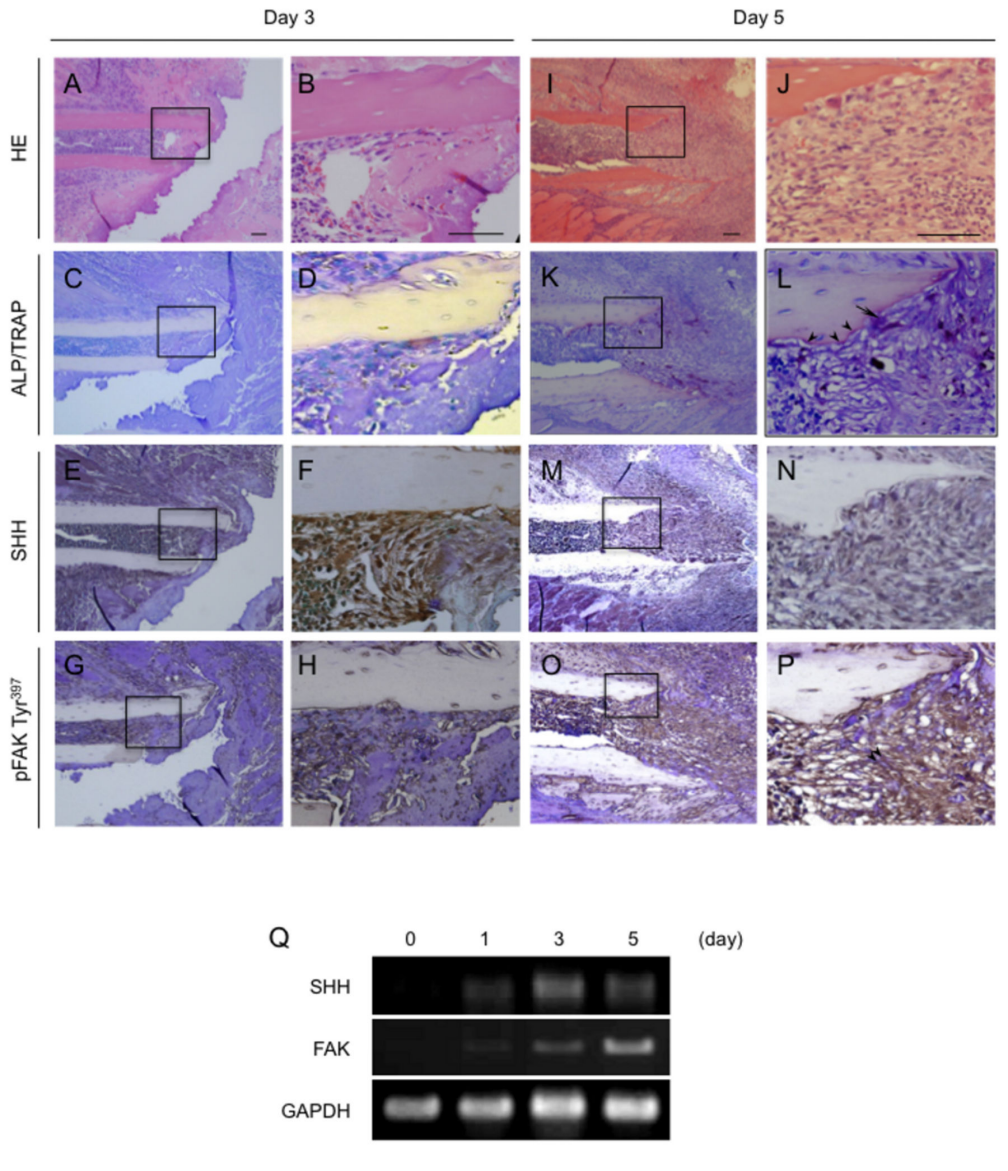
Histological appearance and localization of SHH and pFAK Tyr^397^ in fractured mouse ribs at the early stage after fracturing. A-P, Photomicrographs show the healing process at 3 (A-H) and 5 (I-P) after fracturing. Sections were stained with HE (A, B, I and J) or for ALP and TRAP (C, D, K, and L), SHH (E, F, M, and N), and pFAK Tyr^397^ (G, H, O, and P). Q, RT-PCR analysis of tissue samples containing ribs and surrounding tissues within 3 mm from the fracture line on days 1, 3, and 5 after the operation. Each right photo showing a histological section is a magnification of the rectangle-delimited area in the corresponding left photo Bar, 100 µm. Arrowheads: osteoblasts. Arrow: osteoclast. Double arrowheads: migrating bone marrow cells. The data from a typical experiment are presented: similar results were obtained in repeated experiments.

**Figure 2:**
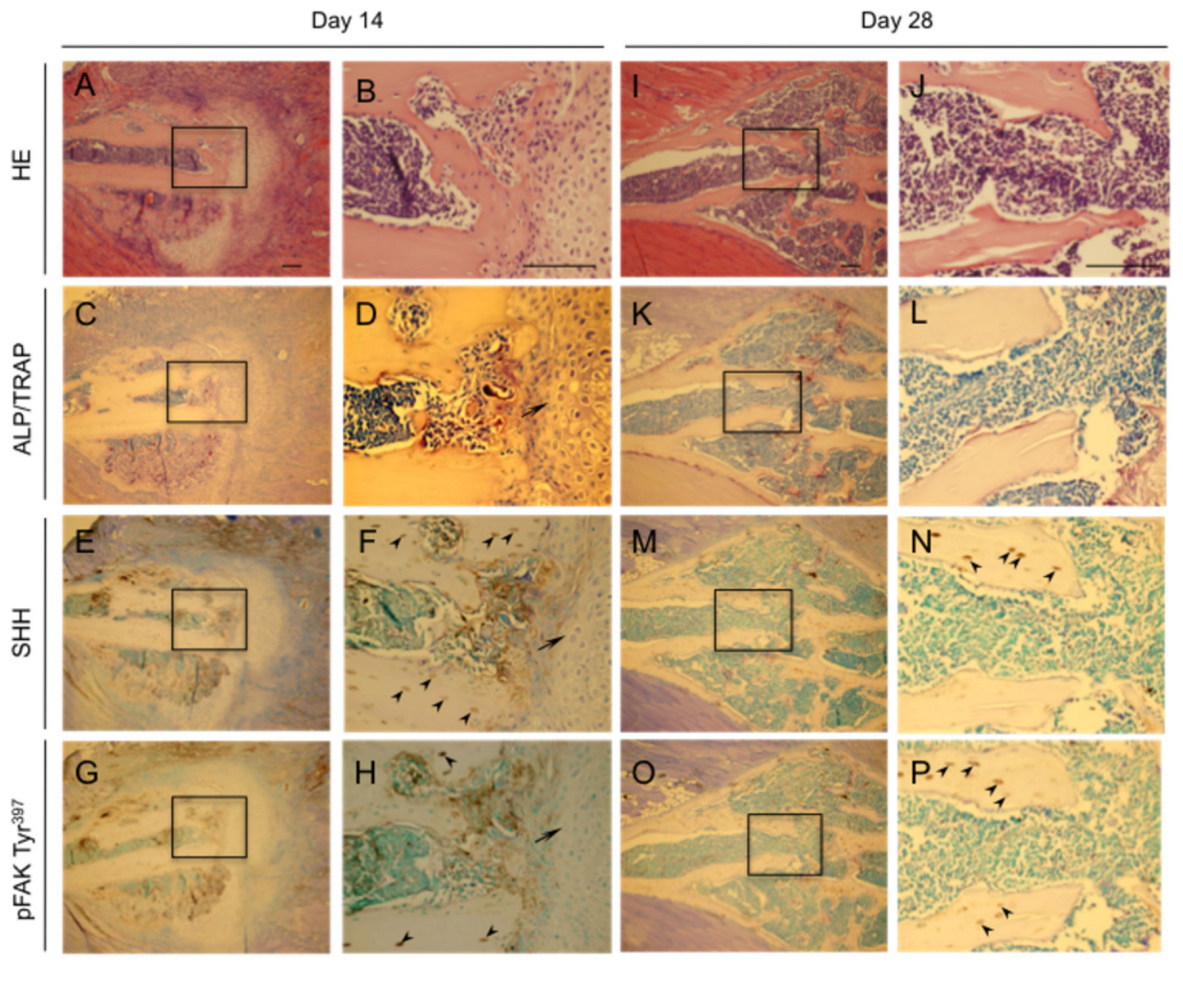
Histological appearance and localization of SHH and pFAK Tyr^397^ in fractured mouse ribs at later stages after fracturing. Photomicrographs show the healing process at 14 (A-H) and 28 (I-P) after fracturing. Sections were stained with HE (A, B, I, and J) or for ALP and TRAP (C, D, K, and L), SHH (E, F, M, and N), pFAK Tyr^397^ and (G, H, O and P). Each right photo is a magnification of the rectangle-delimited area in the corresponding left photo. Bar, 100 µm. Arrowheads: hypertrophic chondrocytes. Arrows: osteocytes. The data from a typical experiment are presented: similar results were obtained in repeated experiments.

We first evaluated the time course of histological changes in a mouse bone fracture model, making observations on days 3, 5, 14, and 28 days after rib fracture. Hematoxylin-stained day 3 sections showed in Figure 1A and B. Neither ALP- nor TRAP-positive cells were found in the fracture site (Figure 1C and D). At that time SHH expression was intense in many of these cells (Figure 1E and F), whereas pFAK Tyr^397^ was weakly expressed in the bone marrow cells (Figure 1G and H). The hematoxylin-stained day 5 sections showed in Figure 1I and J. Staining for ALP and TRAP revealed ALP- (light purple) and TRAP- (dark purple) positive cells at the margin of the cortical bone (Figure 2K and L). SHH expression in the bone marrow cells was decreased compared with that at day 3 (Figure 1M and N), whereas pFAK Tyr^397^ expression was up-regulated in the migrating bone marrow cells, including osteoblasts, at the fracture site (Figure 1O and P). Similar results were also obtained by Immunofluorescence double staining using osteoblast marker osteopontin and pFAK Tyr^397^ antibodies (Figure S1). As shown in Figure S1, osteopontin positive osteoblasts at the bone surface were highly express pFAK Tyr^397^. We next confirmed the time course of changes in SHH and FAK mRNA expression in the fractured mouse rib. The expression of both mRNAs started to increase on day 1 and peaked on day 3 (SHH) or day 5 (FAK; Figure 1Q). Hematoxylin stained day 14 sections showed in Figure 2A and B. ALP-positive osteoblasts (light purple) and TRAP-positive osteoclasts (dark purple) were detected next to hypertrophic chondrocytes at the rib-fracture site (Figure 2C and D). SHH (Figure 2E and F) and pFAK Tyr^397^ (Figure 2G and H) were expressed in the ALP-positive osteoblast near the hypertrophic chondrocytes and in the osteocytes near the fracture site. The hematoxylin-stained day 28 sections showed in Figure 2I and J. By this time ALP- positive osteoblasts were observed at the surface of new trabecular bone that had been formed by endochondral ossification (Figure 2K and L). SHH and pFAK Tyr^397^ were expressed in the osteocytes in the new bone on day 28 (Figure 2M-P).

### FAK is stimulated by SHH in MC3T3-E1 cells

**Figure 3:**
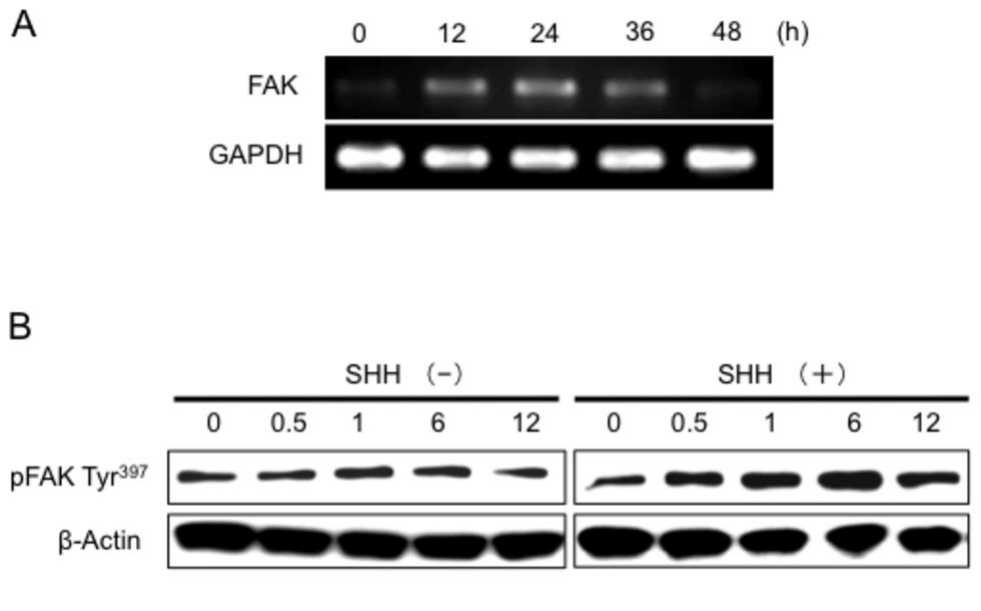
Effect of SHH on the FAK expression in MC3T3-El cells. A, RT-PCR analysis of FAK mRNA expression in MC3T3-El cells after treatment with 500 ng/ml SHH. B, Immunoblot analysis of FAK Tyr^397^ in MC3T3-El cells after incubation with or without 500 ng/ml SHH. The data from a typical experiment are presented: similar results were obtained in repeated experiments.

To determine whether FAK expression would be regulated by SHH in osteoblasts *in vitro*, we used RT-PCR and immunoblot analysis to examine FAK and pFAK Tyr^397^ expression in MC3T3-E1 cells. As shown in Figure 3A, FAK mRNA expression was increased at 12 h after the addition of 500 ng/ml SHH, reaching its maximum at 24 h; whereas that of pFAK Tyr^397^ started to increase at 0.5 h after the start of incubation with 500 ng/ml SHH compared with its expression with vehicle treatment (Figure 3B).

### Establishment of FAK knockdown in MC3T3-E1 cells

**Figure 4:**
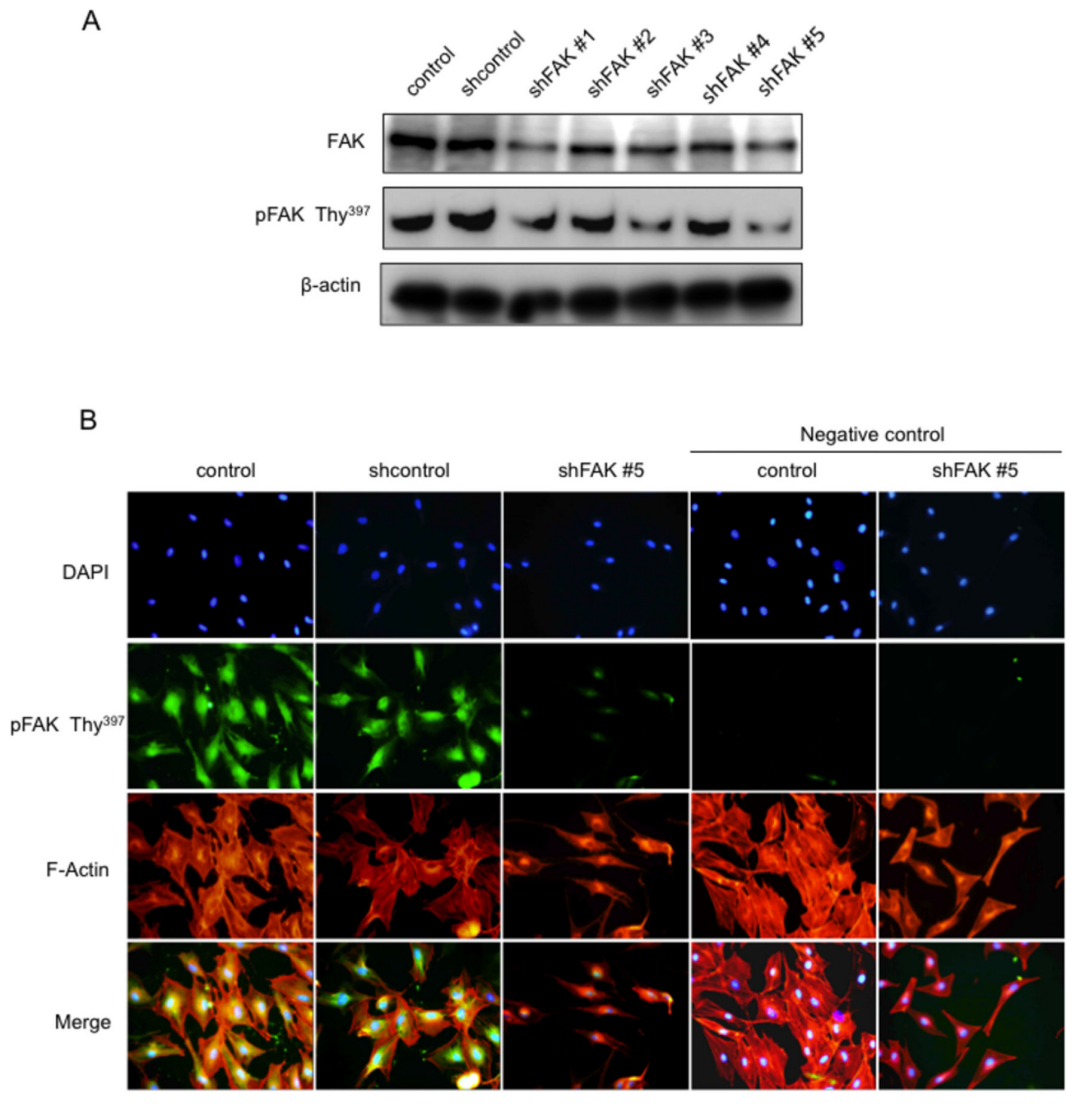
Effect of FAK knockdown by short-hairpin FAK RNAs on MC3T3-El cells and on their morphology. A, Immunoblot analysis of FAK and pFAKTyr^397^ expression in short-hairpin FAK RNA (shFAK #1-#5)-infected MC3T3-El cells. Control, non-infected MC3T3-E1 cells. Shcontrol, control shRNA-infected group. B, Immunofluorescence staining for pFAK Tyr^397^ (green) and F-actin (red) and 4’6’-diamidino-2-phenylindole staining (blue) in control, shcontrol and shFAK#5 RNA-infected MC3T3-El cells. Negative control group was treated with antibody dilution buffer minus pFAKTyr^397^ antibody. The data from a typical experiment are presented; similar results were obtained in repeated experiments.

To analysis the role of FAK in osteoblasts *in vitro*, we infected MC3T3-E1 cells with 5 separate and distinct shRNA lentiviral vectors targeting FAK. As shown in Figure 4A, the expression of both FAK and pFAK Tyr^397^ was suppressed in the shFAK #1-#5-infected groups (shFAK #1-#5) compared with its level in the non-infected MC3T3-E1 cells (control) and in the control shRNA-infected group (shcontrol) (approximately 70% and 84% reduction for FAK, and 81% and 89% reduction for FAK Tyr^397^ in the shFAK #3 and #5 cells individually.) To explore the structural changes in the cells of the shFAK-infected MC3T3-E1 cells, we used fluorescence microscopy to observe the localization of actin and pFAK Tyr^397^ and visualized their nuclei with 4’6’-diamino-2-phenylindole. As shown in Figure 4B, the control and shcontrol MC3T3-E1 cells maintained the normal structure of their actin fibers and expressed pFAK^397^ ; on the other hand, the actin fiber structure formation and pFAK Tyr^397^ expression was dramatically inhibited in the cells infected with shFAK #5 MC3T3-E1 cells. To confirm the specificity of pFAK Tyr^397^ antibody, immunofluorescence staining was performed with the antibody dilution buffer minus pFAK Tyr^397^ antibody. The Immunofluorescence was not observed in the negative control group (Figure 4B).

### Effect of shFAK on the proliferation, adhesion, and migration of MC3T3-E1 cells in the presence or absence of SHH

**Figure 5:**
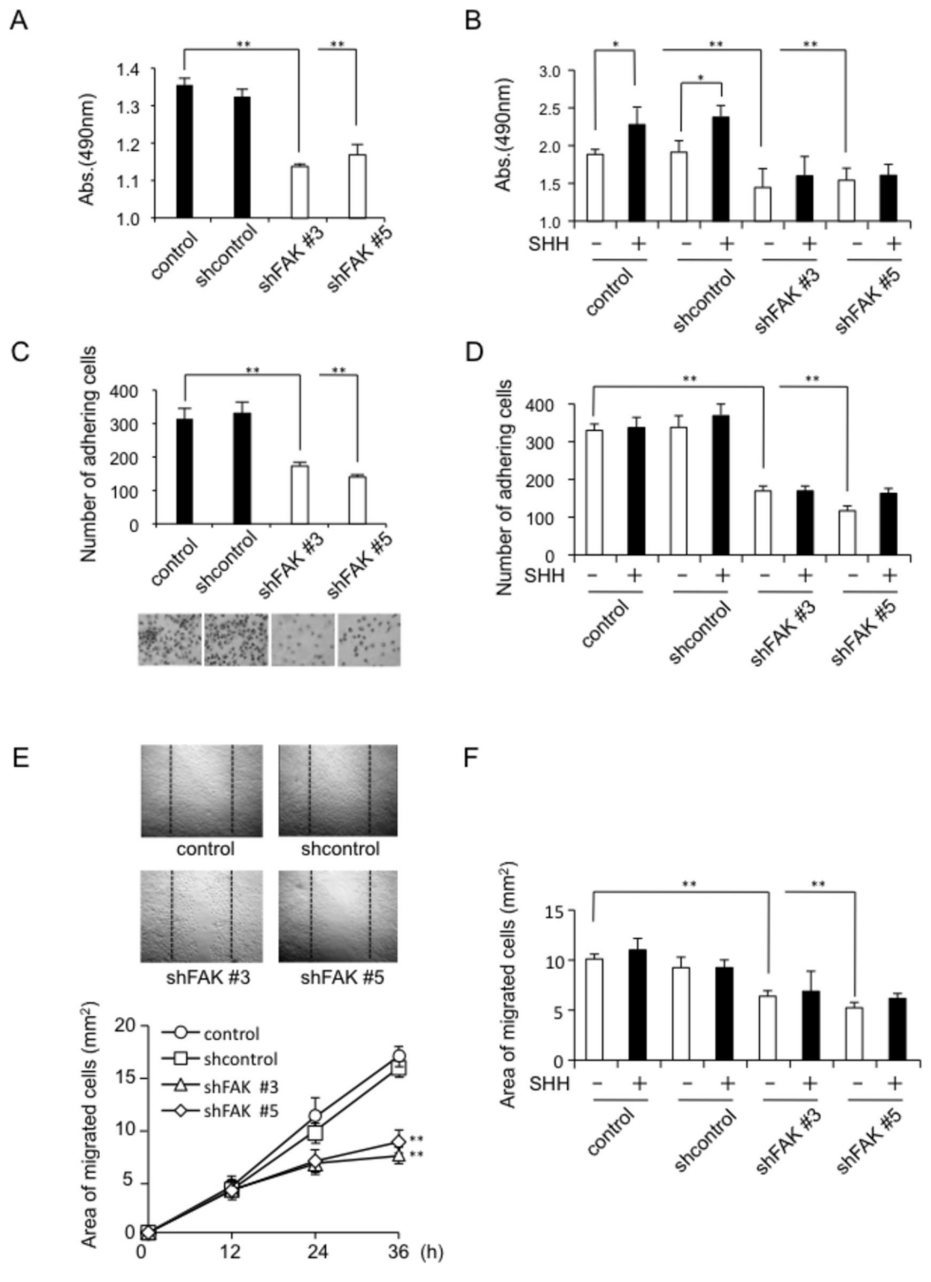
Effect of SHH on the proliferation, adhesion, and migration of FAK knockdown MC3T3-El cells. A, Suppression of growth in shFAK-infected MC3T3-El cells (n = 5). B, Effect of SHH on the proliferation of control or shFAK-infected MC3T3-El cells (n = 5). C, Suppression of cell adhesion of shFAK-infected MC3T3-El cells (n = 5). D, Effect of SHH on the adhesion of control or shFAK-infected MC3T3-El cells (n = 5). E, Suppression of cell migration by shFAK-infected MC3T3-El cells (n = 5). F, Effect of SHH on the migration of control or shFAK-infected MC3T3-El cells after 36 h treatment (n = 5). The data from a typical experiment are presented: similar results were obtained in three separate experiments. Data present mean ± SD. Statistical significance was defined as * *P* < 0.05 and ** *P* < 0.01.

To elucidate the role of FAK involvement in SHH-mediated effects on osteoblasts, we performed proliferation, adhesion, and migration assays using shFAK- infected MC3T3-E1 cells. As shown by the results of the MTS assay in Figure 5A, in the absence of SHH the shFAK #3 and shFAK #5 cells showed significantly suppressed proliferation compared with control and shcontrol groups. SHH stimulated MC3T3-E1 cell proliferation in control and shcontrol cells, whereas it was ineffective on shFAK#3 and shFAK#5 cells (Figure 5B). Figure 5C shows microscopically observed cells attached on the plastic substratum. The shFAK #3 and shFAK #5 cells displayed a round and flattened morphology. Also, they showed suppressed attachment to the plastic dish (Figure 5C). However, SHH had no effect on the adhesion of control, shcontrol, shFAK#3 or shFAK#5 cells (Figure 5D). As shown in Figure 5E, microscopic observations and determination of the area occupied by migrating cells cultured for 36 h after scratching revealed that shFAK cells showed significantly suppressed migration compared with control and shcontrol groups. SHH did not significantly affect the migration of either control or that of shFAK#3 and shFAK#5 cells for 36 h (Figure 5F).

### Effect of shFAK on MC3T3-E1 cell differentiation

**Figure 6:**
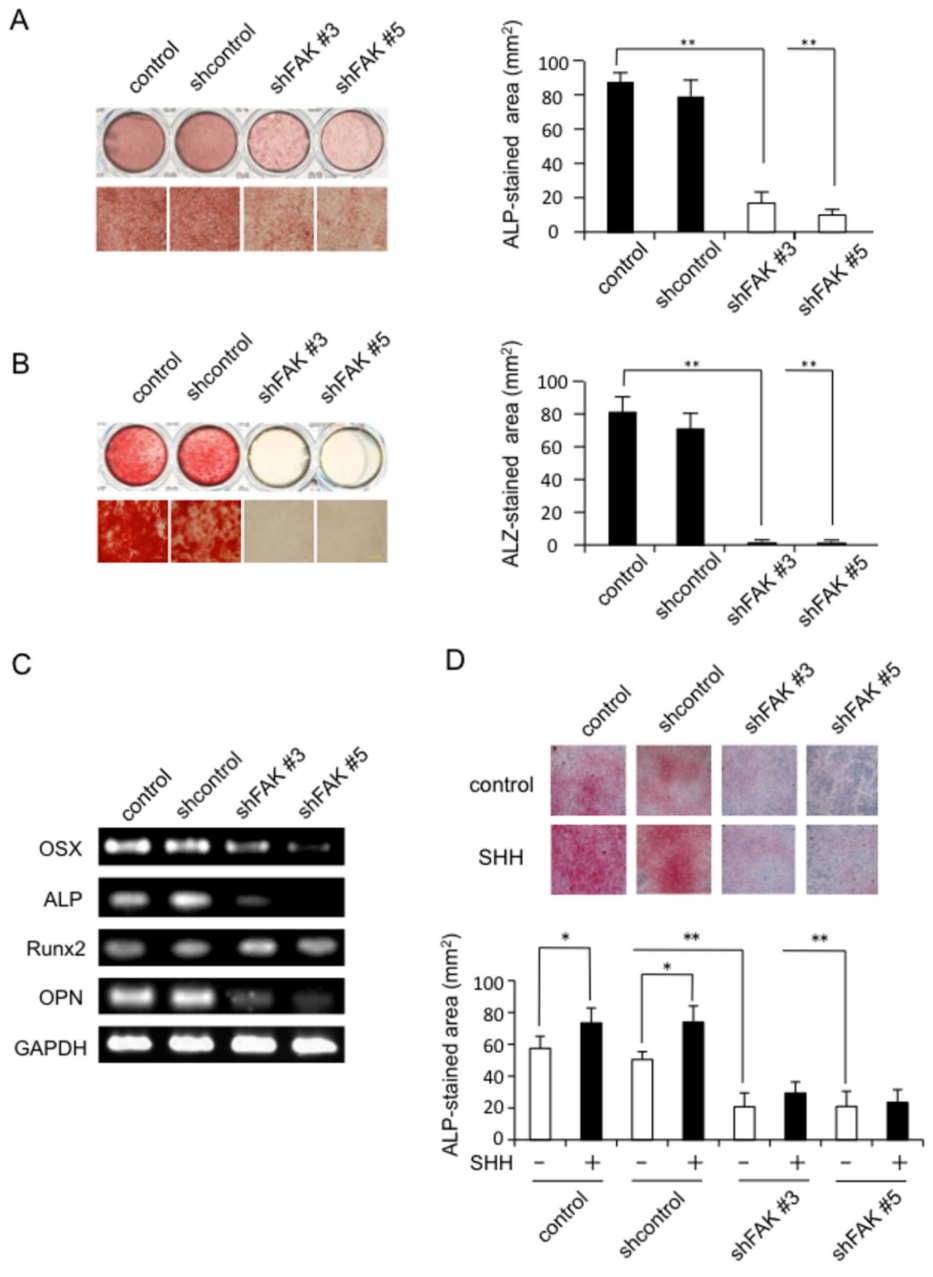
Effect of shFAK and SHH on MC3T3-El cell differentiation. A, Suppression of ALP staining in shFAK-infected MC3T3-El cells (n = 5). B, Suppression of Alizarin red staining in shFAK-infected MC3T3-El cells (n = 5). C, RT-PCR analysis of osterix (OSX), ALP, Runx2, and osteopontin (OPN) mRNA expression in control and shFAK-infected MC3T3-El cells. D, Effect of SHH on the ALP staining of shFAK-infected MC3T3-El cells (n = 5). The data from a typical experiment are presented: similar results were obtained in three separate experiments. Data present mean ± SD. Statistical significance was defined as * *P* < 0.05 and ** *P* < 0.01.

To analyze the effect of shFAK on osteoblast differentiation, we performed ALP and Alizarin red staining of MC3T3-E1 cells that had been grown in medium containing 10% FBS, 50 µg/ml ascorbic acid, and 10 mM β-GP for 5 days. As shown in Figure 6A, shFAK #3 and shFAK #5 cells significantly suppressed ALP staining and the area of ALP-positive cells compared with those for the control and shcontrol groups. In addition, the mineralization effected by shFAK #3 and shFAK #5 cells was completely suppressed compared with that for the control and shcontrol group at 10 days after the start of differentiation (Figure 6B).

To confirm the diminished expression of osteoblast differentiation genes in FAK knockdown cells, we stimulated the cells with 50 µg/ml ascorbic acid and 10 mM β-GP for 5 days, and then performed RT-PCR analysis. As shown in Figure 6C, shFAK#3 and shFAK#5 cells displayed downregulated expression of osterix, ALP, and osteopontin mRNAs whereas Runx2 mRNA expression was the same in all groups. SHH (500 ng/ml) significantly increased the number of ALP-positive cells in both control and shcontrol cells cultured for 5 days in medium containing 10% FBS, 50 µg/ml ascorbic acid, and 10 mM β-GP. However, SHH did not increase the area of ALP-positive cells in shFAK#3 and shFAK#5 cultures (Figure 6D).

### FAK and SHH signalings are involved in PTHrP-stimulated osteoblast-mediated formation of osteoclasts

**Figure 7:**
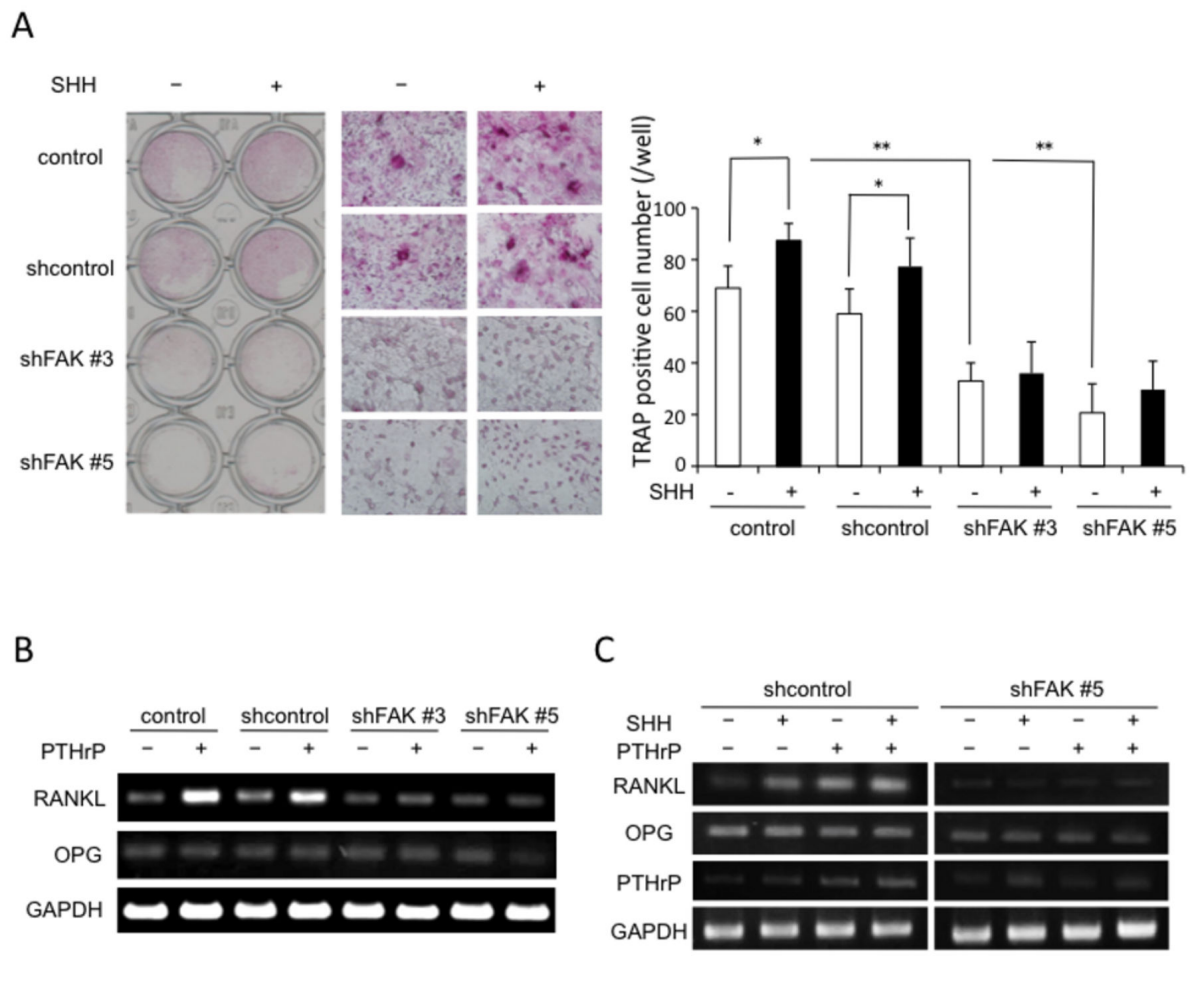
Suppression of osteoclast formation in PTHrP-stimulated co-culture system comprising control MC3T3-E1 cells or shcontrol- or shFAK-infected MC3T3-El cells and murine CD11b^+^ bone marrow cells. A, SHH stimulated an increase in the number of TRAP-positive cells in the presence of 10 nM PTHrP only in co-cultures of control or shcontrol-infected MC3T3-El cells and CD11b^+^ cells (n = 5). Significant differences between the groups are indicated by the brackets: * *P* < 0.05 and ** *P* < 0.01. B and C, RT-PCR analysis of RANKL, OPG and PTHrP expression levels in MC3T3-El cells with or without 10 nM PTHrP or 500 ng/ml SHH. The total RNA was extracted 24 h after the start of treatment. The data from a typical experiment are presented: similar results were obtained in three separate experiments.

Osteoblast progenitors in bone and bone marrow produce PTHrP, which acts through type 1 PTH receptor (PTHR1)-positive preosteoblasts to support hematopoietic precursors to promote increased osteoclast formation through the production of RANKL [22]. Previously we reported that SHH alone has little effect on osteoclastogenesis, but stimulates osteoclast formation in the presence of PTHrP through the upregulation of RANKL in bone marrow stromal cells [10]. To confirm the effect of shFAK and SHH in osteoblasts on PTHrP-induced osteoclast formation, we used a co-culture system comprising MC3T3-E1 cells and CD11b^+^ bone marrow cells as osteoclast precursor cells in the presence of 10 nM PTHrP with or without 500 ng/ml SHH. As shown in Figure 7A, PTHrP-treated shFAK#3 or shFAK#5 cells did not support the formation of TRAP-positive osteoclasts in the presence or absence of SHH compared with control and shcontrol co-culture groups, in which their formation was supported. SHH stimulated osteoclast formation in the control and shcontrol co-culture groups, but not in the shFAK#3 or shFAK#5 co-culture groups. Next, we examined the expression of RANKL and OPG mRNA in MC3T3-E1 cells incubated with or without 10 nM PTHrP (Figure 7B). RT-PCR analysis showed that PTHrP stimulated the expression of RANKL mRNA in control and shcontrol cells, but not in the shFAK#3 or shFAK#5 cells. On the other hand, the expression of OPG mRNA was not changed after the 10 nM PTHrP treatment of either of the controls or of the shFAK cells. Further, to define the role of FAK in SHH-mediated regulation of osteoclast differentiation, we examined the effects of SHH on the expression of RANKL, OPG and PTHrP mRNA in shcontrol or shFAK #5 cells. Combination treatment of 500 ng/ml SHH and 10 nM PTHrP increased the expression of RANKL and PTHrP mRNA more than when separately in shcontrol cells (Figure 7C). However, SHH did not change the expression of RANKL and PTHrP mRNA in the presence of PTHrP in shFAK #5 cells (Figure 7C).

## Discussion

Fracture healing is a complex event in which bone regeneration involves processes associated with normal bone development, including not only cartilage and bone formation, but also endochondral resorption and bone remodeling. Osteogenesis appears to be induced almost immediately after a fracture, as evidenced by the strong induction of type I collagen followed by osteocalcin expression during the early time point starting from day 3 post fracture [23]. It has been reported that FAK can be activated during the osteogenesis process induced by mechanical stretching or high stiffness of ECM substrates [24,25]. On days 1-5, i.e., during the early stage of fracture healing, dynamic regulation of osteoblast cell migration, attachment, and proliferation results in FAK Tyr^397^ phosphorylation in cells at the fractured cortical bone surfaces. In fact, it has been demonstrated that FAK activity is higher in cells seeded on harder surfaces than in those on a soft substratum [26]. FAK is continuously activated during the differentiation process from mesenchymal stem cells toward osteoblasts when this process is induced by bone-inducing agents [27]. This finding is consistent with our present results showing that pFAK Tyr^397^ was not only expressed in the osteoblasts on day 5 after a fracture, but also expressed in the osteocytes at the fracture site on days 14 and 28. It is possible that FAK activity can enhance the bone cell-matrix contacts and promote the mechanosensitivity of the cells toward the hard bone tissue.

Osteoblast migration and proliferation in response to growth factors play a fundamental role in the process of fracture healing [28]. Dynamic processes at work in osteoblasts are required for the coordinated formation and disassembly of focal adhesions, and FAK in osteoblasts is a key component of the signal transduction pathways triggered by fracture. Previous studies suggest that integrin β1-mediated expression of FAK regulates osteoblast behaviors [29], which play an important role in progression in and migration of these cells [30]. The major auto phosphorylation site of FAK Tyr^397^ is absolutely required for enhancing cell migration [31]. Our results suggest that FAK played an important role in the processes of osteoblast adhesion and migration, as FAK Tyr^397^ was expressed in the osteoblasts at the dynamic remodeling site of cortical bone after the fracture. Inhibition of FAK signaling results in reduced cell motility [15], whereas enhancing FAK signaling increases cell migration [32]. Also, extracorporeal shock-wave treatment, a non-invasive but promising method for the treatment of bone fractures, promotes osteoblast adhesion and migration via the induction of the integrin β1 molecule, which is involved in the phosphorylation of FAK Tyr^397^ [19]. It was reported that the hedgehog signaling pathway is related to the mobility and migration of several types of cells [33]. Recent study suggest that SHH signaling induces cell migration through FAK Tyr^397^ signaling pathway [34]. On the other hand, ectopic expression of SHH in the caudal neural tube blocks β1 integrin activation, a mechanism essential for cell-matrix adhesion [35]. Although pFAK Tyr^397^ was presently shown to be activated by SHH, we did not find a significant effect of SHH on osteoblast cell adhesion or migration. Our results indicate that FAK activity involved in the cell adhesion and migration were partially regulated by SHH signaling in osteoblasts. Our data also suggest that SHH derived from bone marrow cells stimulated osteoblast proliferation through FAK signaling. This possibility is consistent with previous studies showing that differentiation of mesenchymal stem cells into osteoblasts is associated with a decrease in hedgehog signaling [36]. Since mesenchymal stem cell differentiation is associated with growth arrest, hedgehog signaling appears to be a candidate for controlling the proliferation of these cells. Furthermore, ectopic expression of GLI1, an effector in the SHH signal transduction pathway, increase integrin β1 levels along with increased proliferation [37]. SHH binds laminin and promotes cell proliferation in an integrin β1-dependent manner [38]. These results suggest that a possible interaction between β1 integrin-mediated FAK activation and SHH occurred in the process of osteoblast proliferation.

In addition, bone-induction medium containing dexamethasone and β-GP can transiently promote FAK Tyr^397^ phosphorylation [39], but its effect on osteoblast differentiation is not clear. Our results showed that suppression of FAK in osteoblasts significantly blocked ALP and Alizarin red staining. The results on osteoblast differentiation suggested that FAK knockdown in osteoblasts led to down-regulation of osterix, a zinc-finger transcription factor that plays a critical role in osteoblast differentiation and bone formation in osteoblasts [40]. FAK siRNA knockdown decreased ALPase activity and blocked osterix transcriptional activity in osteogenic cells [25]. Previous studies have indicated that SHH stimulates osteoblast differentiation *in vivo* and *in vitro* [7,8]. Knocking down of osterix using siRNA totally blocked SHH-induced ALP activity in MC3T3-E1 cells, while knocking down of Runx2 only partially blocked SHH-induced ALP activity, indicating that SHH mediates early osteoblast differentiation mainly through upregulation of osterix expression without induction of Runx2 [41]. In this study, we showed that SHH stimulated the ALP activity in MC3T3-E1 cells in the presence of 10% FBS, whereas it did not do so in FAK knockdown osteoblasts. These results indicate that FAK-regulated osterix expression might be the master regulator during the early stage of SHH-induced osteoblast differentiation. However, further studies are needed to clarify the regulation of FAK in the later stage of osteoblast differentiation, in which osterix can be induced by SHH in both Runx2-dependent and -independent manners [41].

During the initial stage of fracture healing, osteoblasts and osteoclasts were observed on the surface of cortical bone at the fracture site. The replacement of cartilage by osteoid tissue, osteoblasts, and osteoclasts were also presented by the hypertrophic chondrocytes (Figure 2D). To better understand the effect of FAK on osteoclast formation, we used a co-culture system consisting of preosteoblastic MC3T3-E1 cells and murine CD11b^+^ bone marrow cells as osteoclast precursor cells. Osteoblasts are essential for osteoclast formation through a cell-to-cell interaction with osteoclast progenitor cells [42]. As expected, SHH significantly increased the number of TRAP-positive CD11b^+^ bone marrow cells in the presence of PTHrP. SHH stimulated the RANKL and PTHrP mRNA expression under the PTHrP treatment in MC3T3-E1 cells. These data are consistent with previous findings that the increased osteoclastogenic capacity of SHH-treated bone marrow stromal cells occurs via increased RANKL expression by SHH in the presence of PTHrP [10]. In the developing growth plate, hedgehog signals regulate PTHrP levels [43] and a similar mode of regulation is operative in MC3T3-E1 cells, where PTHrP expression and osteolysis is driven at least in part by Gli2 [44,45]. However, we did not detect TRAP-positive cells among FAK knockdown MC3T3-E1 cells with or without SHH. These results suggest that SHH stimulated osteoclast formation by up-regulating osteoblast function under the FAK signaling. In addition to the increased expression of RANKL by PTHrP secreted by osteoblasts, we found no effect of the PTHrP pathway on osteoclastogenesis in the absence of FAK signaling. These findings are similar to earlier ones indicating that a specific inhibitor of pFAK Tyr^397^ suppresses the support of osteoclastogenesis by bone stromal cells via not OPG mRNA regulation but rather RANKL mRNA down-regulation [20].

In summary, we investigated spatiotemporal expression of SHH and FAK Tyr^397^, and the function of SHH in osteoblasts by using a shFAK lentivirus infection model. Our data suggested that SHH signaling was activated in osteoblasts at the dynamic remodeling site of a bone fracture and regulated their proliferation and differentiation, as well as osteoclast formation, via FAK signaling.

## Supporting Information

Figure S1 **Immunofluorescence staining for osteopontin (green); pFAKTyr^397^ (red) and 4’6’–diamino-2-phenylindole staining (blue) in the fractured site on day 5.** Dotted line indicate the bone surface at the fracture site.
(TIF)
