# Supplementary figures and images for: Sonic Hedgehog Regulates Osteoblast Function by Focal Adhesion Kinase Signaling in the Process of Fracture Healing
Source: PLoS One. 2013 Oct 4;8(10):e76785. doi: 10.1371/journal.pone.0076785 (PMC3790742; doi:10.1371/journal.pone.0076785)

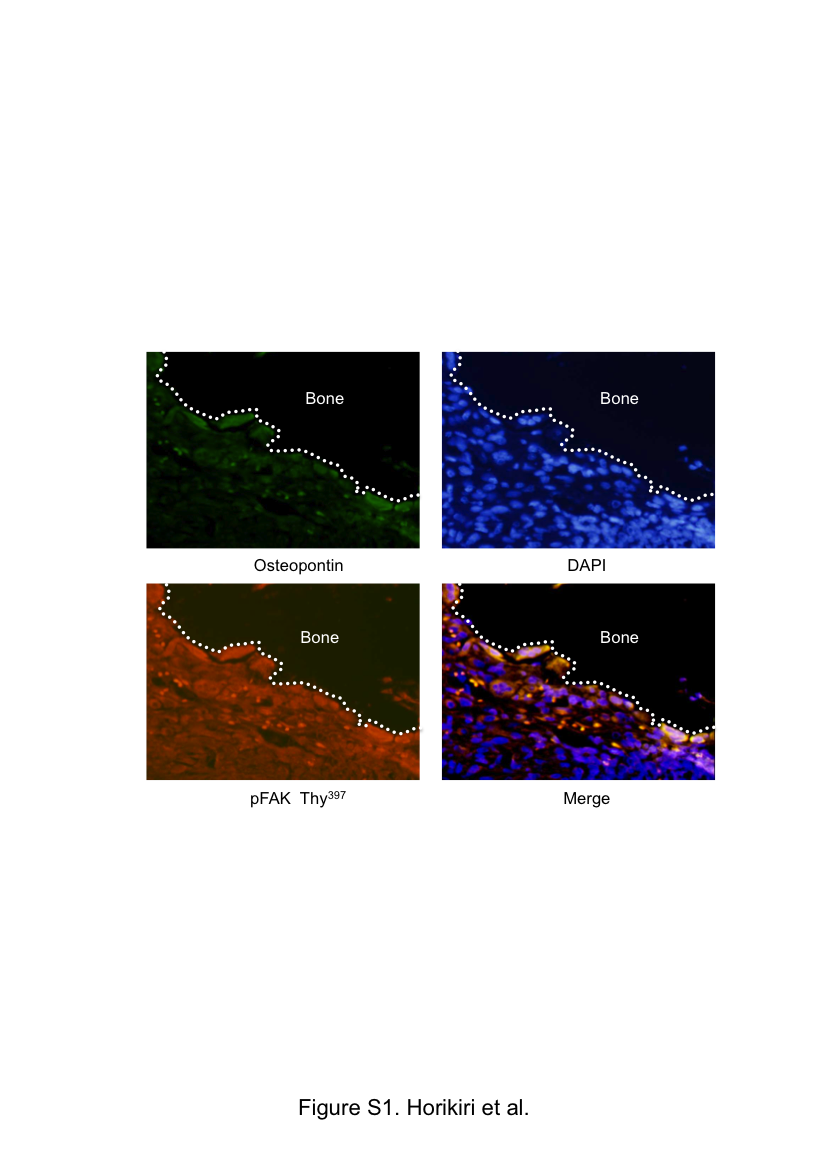

Supplement: Figure S1 — Immunofluorescence staining for osteopontin (green); pFAKTyr397 (red) and 4’6’–diamino-2-phenylindole staining (blue) in the fractured site on day 5. Dotted line indicate the bone surface at the fracture site. (TIF) [file pone.0076785.s001.tif]
